# The inflammatory marker SIRI acts as a statistical mediator between ApoB/ApoA1 ratio and atrial fibrillation, and combined biomarkers show higher discriminative ability for the presence of atrial fibrillation than single indicators

**DOI:** 10.3389/fcvm.2026.1915694

**Published:** 2026-08-13

**Authors:** Chenyang Zheng, Meixue Jia, Bin Ning

**Affiliations:** Department of Cardiovascular Medicine, Anhui Medical University Affiliated Fuyang People’s Hospital, Fuyang People’s Hospital, Fuyang, Anhui, China

**Keywords:** ApoB/ApoA1, atrial fibrillation, inflammation, lipid metabolism, SIRI

## Abstract

**Objective:**

This study aimed to investigate whether the systemic inflammation response index (SIRI) acts as a mediator between the apolipoprotein B/A1 ratio and atrial fibrillation, and assess the combined discriminative ability of the two indicators for identifying AF.

**Method:**

In this retrospective single-center analysis, a final cohort of 638 individuals was enrolled for analysis. Among them, persistent atrial fibrillation (PeAF) was diagnosed in 212 cases and paroxysmal AF (PAF) in 212. The reference group comprised 214 age-matched inpatients without AF. To investigate the associations among SIRI, the ApoB/ApoA1, and AF risk, we used a multivariable analysis. Additionally, fully adjusted logistic regression models with restricted cubic spline (RCS) terms were employed to explore potential nonlinear relationships between SIRI, the ApoB/ApoA1, and AF presence. Spearman analysis was used to assess their monotonic relationship. Mediation analysis was conducted with ApoB/ApoA1 as the exposure and SIRI as the mediator, with AF as the outcome. ROC analyses were performed to evaluate the discriminative accuracy of ApoB/ApoA1, SIRI, and their combination for AF detection. DeLong's test was used to compare the AUCs. Sensitivity, specificity, and Brier score were calculated across multiple cutoff points, with the Youden index identifying the optimal threshold. Finally, we performed a subgroup analysis stratified by the use of lipid-lowering drugs to evaluate the potential effect modification by lipid-lowering therapy.

**Result:**

The mediation analysis indicated that SIRI served as a partial statistical mediator of the inverse ApoB/ApoA1–AF relationship. The ROC analysis showed AUCs of 0.728 for ApoB/ApoA1, 0.760 for SIRI and 0.799 for the combined model. When contrasted with ApoB/ApoA1 alone, the ApoB/ApoA1 + SIRI combination achieved a significantly higher AUC (DeLong test, *P* < 0.001). Compared with SIRI alone, the integrated model also yielded a notably larger AUC (DeLong test, *P* = 0.002). The ApoB/ApoA1 + SIRI model exhibited the highest AUC, along with improved specificity, the lowest Brier score, and positive discriminative value. Besides, these findings are not affected by lipid-lowering drug use. However, given the cross-sectional design, these finding reflects statistical interdependence rather than a causal sequence.

**Conclusion:**

Elevated SIRI and reduced ApoB/ApoA1 were significantly associated with higher odds of AF. The statistical mediation effect of SIRI suggests a potential interrelationship among these variables. The combined model outperformed all others in discriminative performance, offering a practical exploratory tool for patient stratification. However, these findings require independent external validation and prospective confirmation.

## 1 Introduction

Apolipoprotein B (ApoB) and Apolipoprotein A1 (ApoA1) are central indicators of lipid metabolism. The ApoB/ApoA1 ratio captures the balance between pro-atherogenic and anti-atherogenic lipoproteins and is comparatively less affected by statin treatment, potentially offering a more precise assessment of how lipid abnormalities influence cardiovascular risk than traditional lipid parameters. Several studies (1–4) have found that ApoB/ApoA1 more effectively identifies risks linked to coronary atherosclerotic disease, myocardial infarction, and ischemic stroke. It is also associated with cardiovascular all-cause mortality and atrial fibrillation. Moreover, a low serum ApoB/ApoA1 ratio may be associated with the onset of AF (5). Nevertheless, evidence exploring the relationship between ApoB/ApoA1, inflammation, and atrial fibrillation remains limited.

Atrial fibrillation (AF) is a prevalent and clinically significant arrhythmia, linked to substantial morbidity and mortality due to complications such as cardiomyopathy, heart failure, and stroke (6). A variety of therapeutic approaches are available, including antiarrhythmic drugs to maintain sinus rhythm, rate-control agents to prevent rapid heart rates, anticoagulants to reduce thromboembolism risk, and catheter ablation to sustain sinus rhythm. Although these strategies can be beneficial, each bears limitations and potential adverse effects, highlighting an unmet need for innovative treatments (7). The involvement of inflammation in AF has been proposed for more than two decades, and a growing body of evidence has accumulated (8, 9). Nevertheless, the benefits of traditional anti-inflammatory therapies for AF have generally been modest and inconclusive. Complete blood count–derived markers of inflammation, such as the systemic inflammatory response index (SIRI), have emerged as novel indicators of systemic inflammatory status. AF is a multifactorial disorder arising from lipid dysregulation, obesity, insulin resistance, chronic inflammation, and genetic factors; despite extensive research and proposed mechanisms, how these factors interact in AF pathogenesis remains unclear. In particular, it is not yet proven whether inflammation contributes to the link between apolipoproteins and AF presence. Accordingly, this study aims to elucidate the complex associations among ApoB/ApoA1, AF presence, and SIRI, and to assess the extent to which inflammation associated with the ApoB/ApoA1–AF relationship, with the goal of providing new insights into the interplay between lipid metabolism, inflammation, and AF.

## 2 Materials and methods

### 2.1 Study population

In this retrospective, single-center analysis, a final cohort of 638 individuals was enrolled for analysis, as depicted in the study flow diagram. Among them, persistent atrial fibrillation (PeAF) was diagnosed in 212 cases and paroxysmal AF (PAF) in 212. The reference group also comprised 214 age-matched inpatients without AF. These subjects were randomly selected from the same Department of Cardiovascular Medicine over an identical recruitment window and were hospitalized for cardiovascular conditions other than AF. We thoroughly reviewed the medical histories of all control subjects and conducted a comprehensive clinical assessment to rigorously exclude any evidence of AF. To minimize selection bias, frequency matching was applied based on age (±5 years) and sex to ensure comparability with the AF groups. Data retrieval for the research was performed on 1 April 2024, and all personal identifiers were stripped from the records during collection to ensure anonymity. Patients were excluded from this study if they had any of the following conditions: acute decompensated heart failure, rheumatic valvular heart disease, hypertrophic cardiomyopathy, congenital heart disease, previous cardiac surgery, hyperthyroidism, active inflammation, liver or kidney dysfunction, acute myocardial infarction, or acute cerebral infarction. In addition, patients with a history of systemic glucocorticoid therapy, immunosuppressant use, or chronic anti-inflammatory drug treatment (e.g., NSAIDs) within the 3 months prior to enrollment were also excluded to avoid potential interference with peripheral blood leukocyte counts and SIRI values.

### 2.2 Clinical data and definitions

We collected demographic and clinical data from patients' electronic hospital records and sorted all data into a customized Excel sheet. Recorded variables included age, gender, use of *β*-blockers, ACE inhibitors and calcium channel blockers, body mass index, lipid-lowering treatment, smoking and drinking habits, hypertension, diabetes, coronary artery disease, blood pressure values, and atrial fibrillation subtypes. Venous blood samples were taken from all participants after overnight fasting, and routine biochemical indexes were measured the next morning. These indexes contained alanine aminotransferase, aspartate aminotransferase, serum creatinine, fasting glucose and total bilirubin. We also recorded inflammatory and lipid indicators, specifically white blood cells, neutrophils, lymphocytes, monocytes, platelets, total cholesterol, triglycerides, HDL-C, LDL-C, ApoA1, ApoB, and ApoB/ApoA1. Every patient received an electrocardiogram and an echocardiogram. Their left atrial dimensions were documented. All control subjects underwent a 24 h Holter monitoring. For with AF, Holter monitoring was performed as part of the standard assessment. Atrial fibrillation was classified based on current guidelines: paroxysmal AF terminates within seven days, whereas persistent AF lasts longer than seven days (10). The systemic inflammatory response index was calculated by multiplying the neutrophil count by the monocyte count and then dividing by the lymphocyte count.

### 2.3 Statistical analysis

We completed all statistical analyses with R software version 4.6.0 (R Foundation for Statistical Computing). For normally distributed data, results are summarized as mean ± SD, while nonparametric data are described using quartiles (25th–75th percentiles). Categorical variables are reported as counts with corresponding percentages. To investigate the associations among SIRI, the ApoB/ApoA1 index, and AF prevalence, a multivariable analysis was performed. In addition, to examine possible nonlinear links of SIRI and the ApoB/ApoA1 with the probability of atrial fibrillation, fully adjusted logistic models incorporating restricted cubic spline terms were fitted. Because both ApoB/ApoA1 and SIRI departed from a Gaussian distribution, the Spearman rank order approach was adopted for evaluating their monotonic association. A mediation framework was then applied, treating ApoB/ApoA1 as the independent variable, SIRI as the intermediary, and AF status as the endpoint. Receiver operating characteristic curve analysis was carried out to gauge the discriminatory ability of ApoB/ApoA1 alone, SIRI alone, and the two markers combined for identifying AF. DeLong's method served to compare the resulting areas under the curve. Over a series of cut-off values, sensitivity and specificity were computed, and the Youden index determined the most suitable threshold. The Brier score was calculated to assess the overall discriminative performance of the model. To evaluate the potential effect modification by lipid-lowering therapy, we performed a subgroup analysis stratified by the use of lipid-lowering drugs. Two-tailed testing was performed throughout, and statistical significance was set at a *P* value below 0.05.

## 3 Result

### 3.1 Baseline characteristic

Table 1 displays the demographic and baseline clinical features of the three groups. Relative to the control subjects without atrial fibrillation, individuals diagnosed with AF (including both paroxysmal and persistent forms) showed a markedly greater frequency of coronary heart disease (CHD). In addition, the AF groups displayed significantly elevated values for fasting plasma glucose (FBG), total bilirubin, AST (aspartate aminotransferase), left atrial dimension (LA), body mass index (BMI), leukocyte count, neutrophil count, monocyte count, the systemic inflammatory response index (SIRI), and serum creatinine (SCR). Moreover, the proportion of patients receiving *β*-blockers, angiotensin-converting enzyme inhibitors (ACEIs), and lipid-lowering agents was significantly higher in the AF cohort. But they had considerably reduced concentrations of high-density lipoprotein cholesterol (HDL), apolipoprotein A1 (ApoA1), triglycerides (TG), apolipoprotein B (ApoB), the ApoB-to-ApoA1 ratio, as well as lower lymphocyte and platelet counts. People with atrial fibrillation tended to be older than those without the arrhythmia. Moreover, among the AF patients, the persistent form was associated with a more advanced age compared with the paroxysmal form. In contrast, the groups did not differ significantly regarding sex distribution, hypertension, diabetes mellitus (DM), smoking status, alcohol consumption, alanine aminotransferase (ALT) levels, or the use of calcium channel blockers (CCBs).

**Table 1:** Comparison of baseline characteristics Among study participants.

Variables	Total (*n* = 638)	non-AF (*n* = 214)	Paroxysmal atrial fibrillation (*n* = 212)	Persistent atrial fibrillation (*n* = 212)	Statistic	*P*
Age, M (Q₁, Q₃)	72.00 (66.00, 78.00)	69.00 (60.00,74.00)	73.00 (68.75,77.25)	74.00 (69.00,80.00)	*χ*² = 56.56	<.001
FBG, M (Q₁, Q₃)	5.28 (4.67, 6.26)	5.09 (4.56,5.77)	5.50 (4.77,6.52)	5.36 (4.69,6.41)	*χ*² = 16.95	<.001
TC, M (Q₁, Q₃)	3.54 (2.90, 4.34)	3.72 (2.86,4.76)	3.44 (3.01,4.13)	3.48 (2.90,4.18)	*χ*² = 6.50	0.039
TG, M (Q₁, Q₃)	1.01 (0.76, 1.38)	1.12 (0.87,1.52)	0.93 (0.68,1.18)	0.92 (0.68,1.31)	*χ*² = 40.33	<.001
HDL, M (Q₁, Q₃)	1.08 (0.91, 1.33)	1.21 (0.95,1.6)	1.04 (0.89,1.23)	1.04 (0.88,1.24)	*χ*² = 34.62	<.001
LDL, M (Q₁, Q₃)	1.78 (1.35, 2.39)	2.04 (1.35,2.71)	1.76 (1.36,2.28)	1.65 (1.33,2.21)	*χ*² = 9.88	0.007
ApoB, M (Q₁, Q₃)	0.76 (0.64, 0.93)	0.92 (0.79,1.10)	0.71 (0.61,0.81)	0.71 (0.60,0.84)	*χ*² = 123.79	<.001
ApoA1, M (Q₁, Q₃)	1.27 (1.14, 1.40)	1.30 (1.17,1.44)	1.22 (1.11,1.36)	1.27 (1.14,1.42)	*χ*² = 13.54	0.001
TotalBilirubin, M (Q₁, Q₃)	16.40 (12.83, 20.30)	14.45 (11.22,18.05)	18.45 (14.38,23.22)	16.95 (13.85,20.27)	*χ*² = 50.75	<.001
ALT, M (Q₁, Q₃)	18.00 (13.00, 25.00)	17.70 (12.83,23.60)	19.20 (13.10,26.63)	17.00 (13.25,24.60)	*χ*² = 2.31	0.315
AST, M (Q₁, Q₃)	21.30 (16.90, 26.60)	20.20 (16.02,24.20)	22.85 (18.70,28.70)	20.80 (17.25,27.50)	*χ*² = 20.97	<.001
BMI, M (Q₁, Q₃)	23.80 (21.50, 26.40)	22.90 (21.00,25.88)	24.40 (21.78,26.80)	24.40 (22.00,27.02)	*χ*² = 14.55	<.001
ApoB/APoA1, M (Q₁, Q₃)	0.61 (0.52, 0.74)	0.70 (0.61,0.87)	0.57 (0.48,0.68)	0.57 (0.49,0.66)	*χ*² = 89.14	<.001
WhiteBloodCellCount, M (Q₁, Q₃)	5.58 (4.64, 6.61)	5.26 (4.55,6.42)	5.70 (4.88,6.64)	5.86 (4.73,6.83)	*χ*² = 8.05	0.018
NeutrophilCount, M (Q₁, Q₃)	3.54 (2.84, 4.42)	3.07 (2.54,3.94)	3.74 (3.02,4.64)	3.85 (3.07,4.72)	*χ*² = 43.15	<.001
LymphocyteCount, M (Q₁, Q₃)	1.39 (1.10, 1.82)	1.62 (1.31,2.04)	1.33 (1.02,1.78)	1.25 (0.98,1.59)	*χ*² = 59.81	<.001
MonocyteCount, M (Q₁, Q₃)	0.38 (0.30, 0.47)	0.35 (0.28,0.43)	0.41 (0.31,0.48)	0.40 (0.31,0.50)	*χ*² = 24.94	<.001
PlateletCount, M (Q₁, Q₃)	182.00 (144.00, 217.00)	193.00 (154.75,231.25)	174.50 (133.00,210.25)	178.00 (146.75,211.25)	*χ*² = 12.50	0.002
SIRI, M (Q₁, Q₃)	0.99 (0.62, 1.45)	0.65 (0.49,0.99)	1.18 (0.76,1.63)	1.18 (0.85,1.72)	*χ*² = 117.37	<.001
LA, M (Q₁, Q₃)	38.00 (33.00, 44.00)	32.50 (30.00, 38.00)	38.00 (34.00, 46.00)	44.00 (38.00, 48.00)	*χ*² = 189.65	<.001
SCR, M (Q₁, Q₃)	64.15 (55.55, 73.27)	61.05 (53.35,69.85)	65.40 (54.60,76.95)	66.10 (58.80,75.35)	*χ*² = 18.49	<.001
Sex, *n*(%)					*χ*² = 0.24	0.888
Female	318 (49.84)	109 (50.93)	103 (48.58)	106 (50.00)		
Male	320 (50.16)	105 (49.07)	109 (51.42)	106 (50.00)		
Hypertension, *n*(%)					*χ*² = 0.51	0.774
No	277 (43.42)	96 (44.86)	93 (43.87)	88 (41.51)		
Yes	361 (56.58)	118 (55.14)	119 (56.13)	124 (58.49)		
CoronaryHeartDisease, *n*(%)					*χ*² = 20.85	<.001
No	411 (64.42)	162 (75.70)	116 (54.72)	133 (62.74)		
Yes	227 (35.58)	52 (24.30)	96 (45.28)	79 (37.26)		
Diabetes, *n*(%)					*χ*² = 1.39	0.500
No	452 (70.85)	156 (72.90)	152 (71.70)	144 (67.92)		
Yes	186 (29.15)	58 (27.10)	60 (28.30)	68 (32.08)		
Smoking, *n*(%)					*χ*² = 1.05	0.591
No	495 (77.59)	171 (79.91)	163 (76.89)	161 (75.94)		
Yes	143 (22.41)	43 (20.09)	49 (23.11)	51 (24.06)		
Drinking, *n*(%)					*χ*² = 1.91	0.384
No	516 (80.88)	176 (82.24)	165 (77.83)	175 (82.55)		
Yes	122 (19.12)	38 (17.76)	47 (22.17)	37 (17.45)		
*β*-blockersUse, *n*(%)					*χ*² = 8.46	0.015
No	563 (88.24)	200 (93.46)	182 (85.85)	181 (85.38)		
Yes	75 (11.76)	14 (6.54)	30 (14.15)	31 (14.62)		
ACEIUse, *n*(%)					*χ*² = 7.31	0.026
No	525 (82.29)	178 (83.18)	184 (86.79)	163 (76.89)		
Yes	113 (17.71)	36 (16.82)	28 (13.21)	49 (23.11)		
CCBUse, *n*(%)					*χ*² = 3.25	0.197
No	531 (83.23)	172 (80.37)	184 (86.79)	175 (82.55)		
Yes	107 (16.77)	42 (19.63)	28 (13.21)	37 (17.45)		
LipidLoweringDrugUse, *n*(%)					*χ*² = 7.05	0.029
No	516 (80.88)	183 (85.51)	173 (81.60)	160 (75.47)		
Yes	122 (19.12)	31 (14.49)	39 (18.40)	52 (24.53)		

ApoA1, Apolipoprotein A1; ApoB, Apolipoprotein B; AF, atrial fibrillation; ALT, alanine aminotransferase; AST, aspartate aminotransferase; BMI, body mass index; ACEI, angiotensin-converting enzyme inhibitor; CCB, calcium channel blocker; FBG, fasting blood glucose; HDL-C, high-density lipoprotein cholesterol; LA, left atrium; LDL-C, low-density lipoprotein cholesterol; SCR, serum creatinine; SIRI, systemic inflammatory response index; TC, total cholesterol; TG, triglycerides.

### 3.2 Logistic regression analysis

ApoB/ApoA1 and SIRI were grouped into tertiles. In both the continuous and tertile-based analyses, higher SIRI levels were associated with a markedly greater likelihood of AF; Table 2 shows a clear dose–response pattern (*P* < 0.001 for all comparisons). By contrast, the ApoB/ApoA1 ratio acted as an independent protective factor against AF, with participants in the top tertile exhibiting progressively lower presence (*P* < 0.05). These relationships remained consistent across crude, partially adjusted, and fully adjusted models. Therefore, both ApoB/ApoA1 and SIRI independently identify AF presence.

**Table 2:** Multivariate regression analysis for associations of SIRI and ApoB/ApoA1 with AF presence.

Variables	OR (95%CI)	*P*	OR (95%CI)	*P*	OR (95%CI)	*P*
SIRI	6.54 (4.29 ∼ 9.98)	<.001	5.70 (3.55 ∼ 9.16)	<.001	6.49 (3.86 ∼ 10.92)	<.001
<0.72	1.00 (Reference)		1.00 (Reference)		1.00 (Reference)	
0.72–1.27	2.94 (1.98 ∼ 4.37)	<.001	2.57 (1.59 ∼ 4.14)	<.001	2.48 (1.50 ∼ 4.10)	<.001
>1.27	12.11 (7.20 ∼ 20.37)	<.001	9.44 (5.15 ∼ 17.32)	<.001	10.91 (5.63 ∼ 21.17)	<.001
ApoB/ApoA1	0.01 (0.00 ∼ 0.03)	<.001	0.01 (0.00 ∼ 0.03)	<.001	0.01 (0.00 ∼ 0.03)	<.001
<0.55	1.00 (Reference)		1.00 (Reference)		1.00 (Reference)	
0.55–0.68	0.52 (0.32 ∼ 0.84)	0.008	0.53 (0.30 ∼ 0.94)	0.030	0.52 (0.28 ∼ 0.94)	0.031
>0.68	0.17 (0.11 ∼ 0.26)	<.001	0.16 (0.09 ∼ 0.28)	<.001	0.14 (0.08 ∼ 0.25)	<.001

OR, odds ratio; CI, confidence interval.

Model1: Crude.

Model2: Adjust: Sex, Age, BMI, LA.

Model3: Adjust: Sex, Hypertension, CoronaryHeartDisease, Diabetes, Smoking, Drinking, *β*-blockersUse, LipidLoweringDrugUse, Age, FBG, BMI, SCR, LA.

### 3.3 RCS analysis

**Figure 1:**
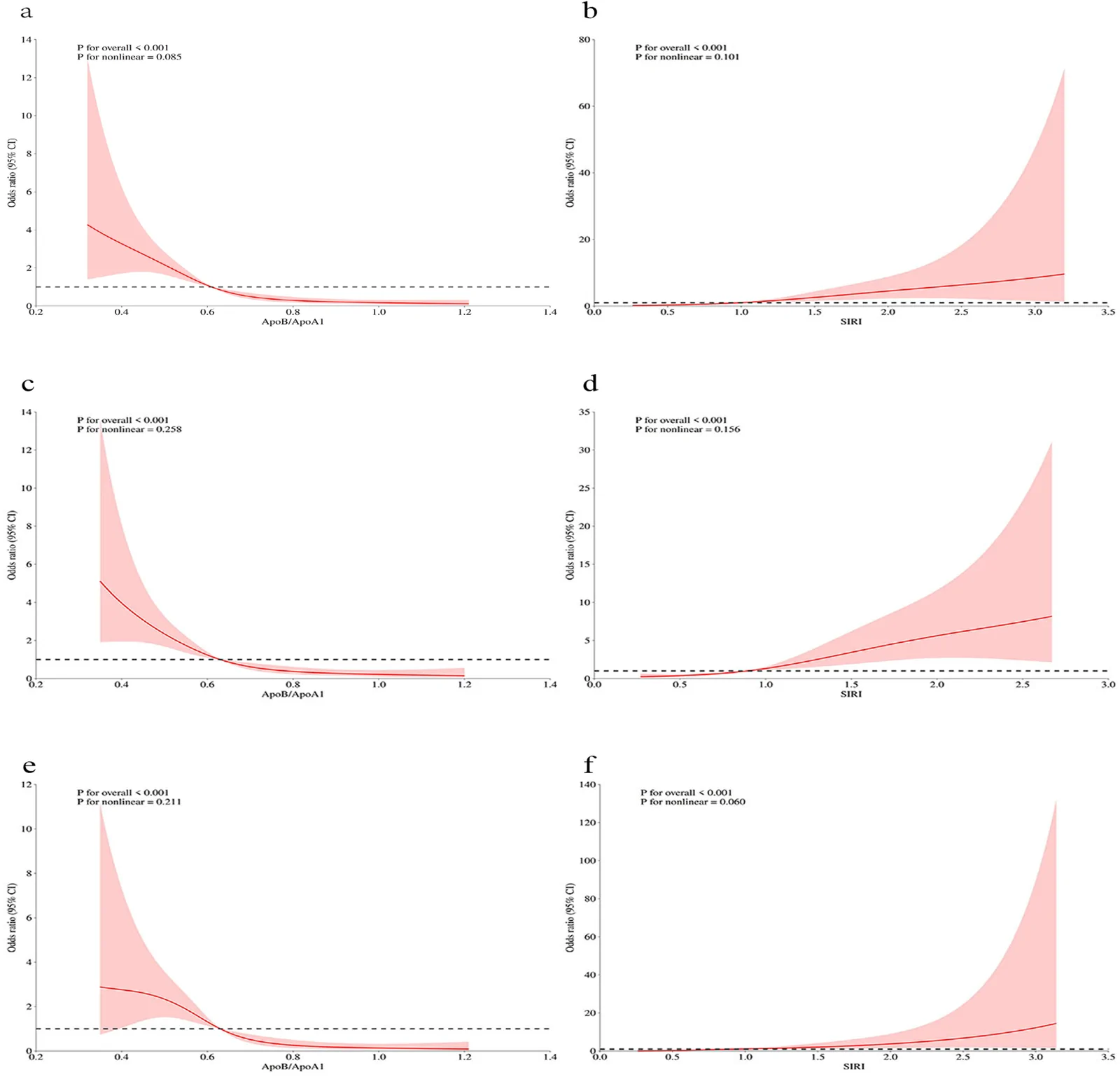
Adjusted associations of SIRI and ApoB/ApoA1 with atrial fibrillation across clinical subgroups (Model 3 adjustment).

Using restricted cubic spline (RCS) regression with covariate adjustment as in Model 3, we identified a positive linear association between SIRI and the presence of AF, and a negative linear association between the ApoB/ApoA1 ratio and the presence of AF (nonlinear *P*-values > 0.001; Figures 1a,b). The same linear patterns were observed in the PAF subgroup (Figures 1c,d), as well as in the PeAF subgroup (Figures 1e,f).

**Figure 3:**
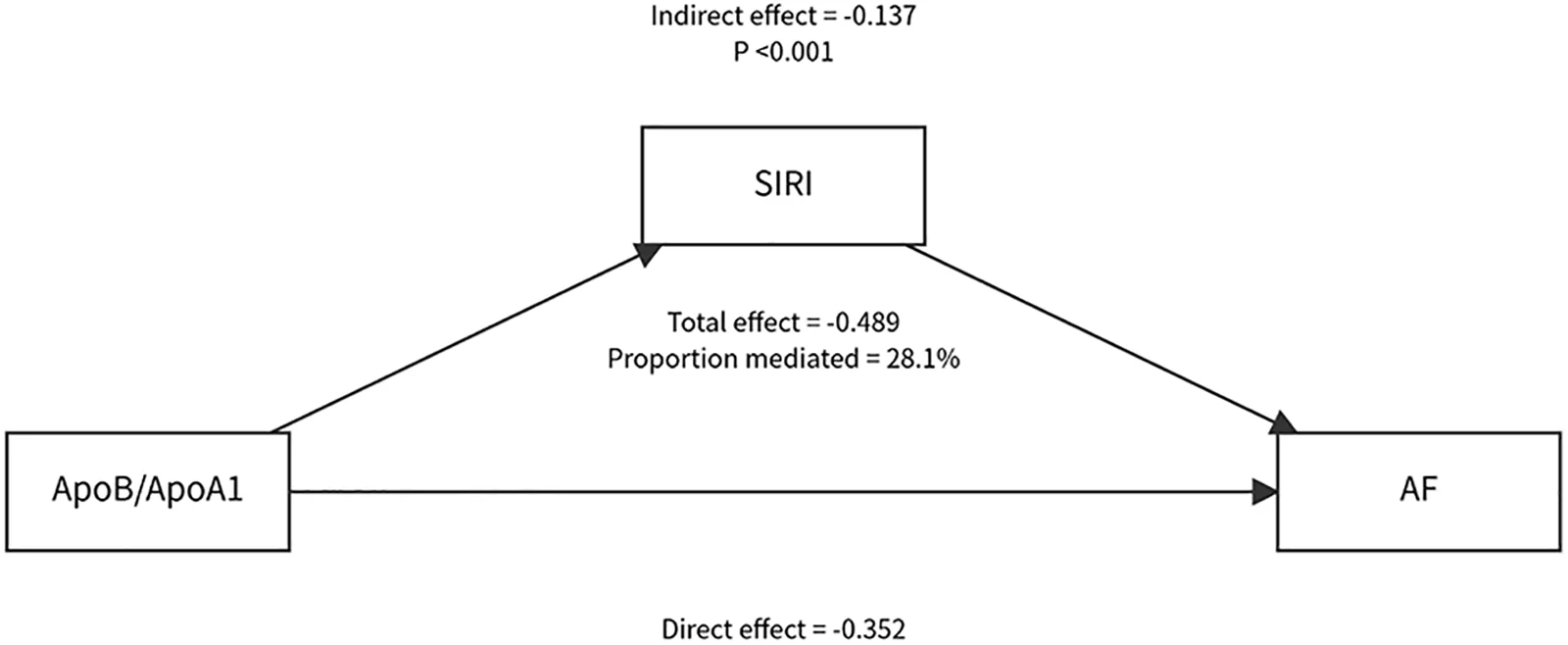
Mediation analysis of the association between SIRI and ApoB/ApoA1 with AF.

### 3.4 Correlation analysis

**Figure 2:**
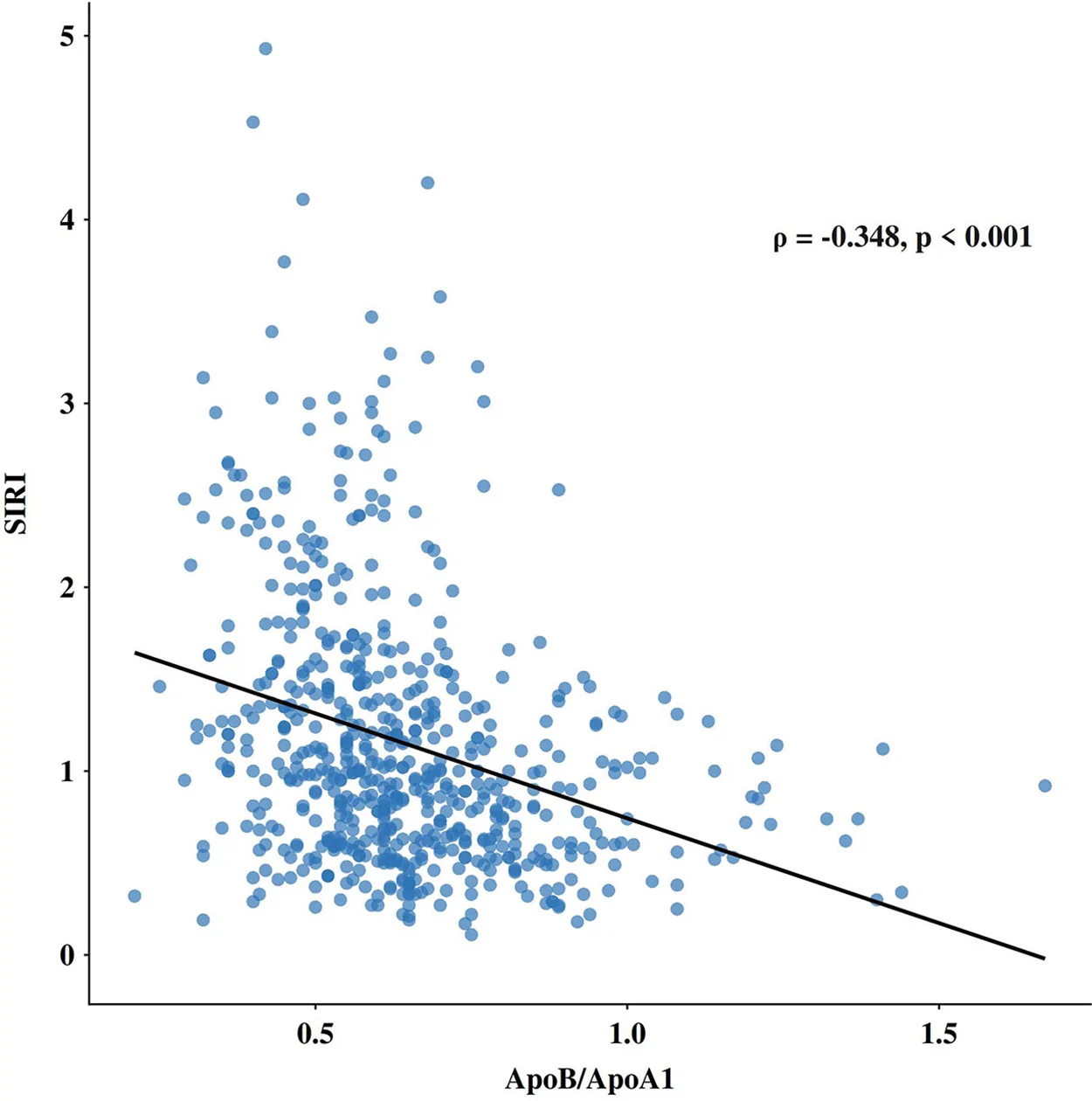
Spearman Correlation Analysis of SIRI and ApoB/ApoA1.

We further explored the relationship between ApoB/ApoA1 and SIRI. Because both measures did not follow a normal distribution, Spearman correlation analysis was employed. As shown in Figure 2, there is a pronounced inverse relation, with a Spearman *ρ* of −0.348 and *P* < 0.001.

**Figure 4:**
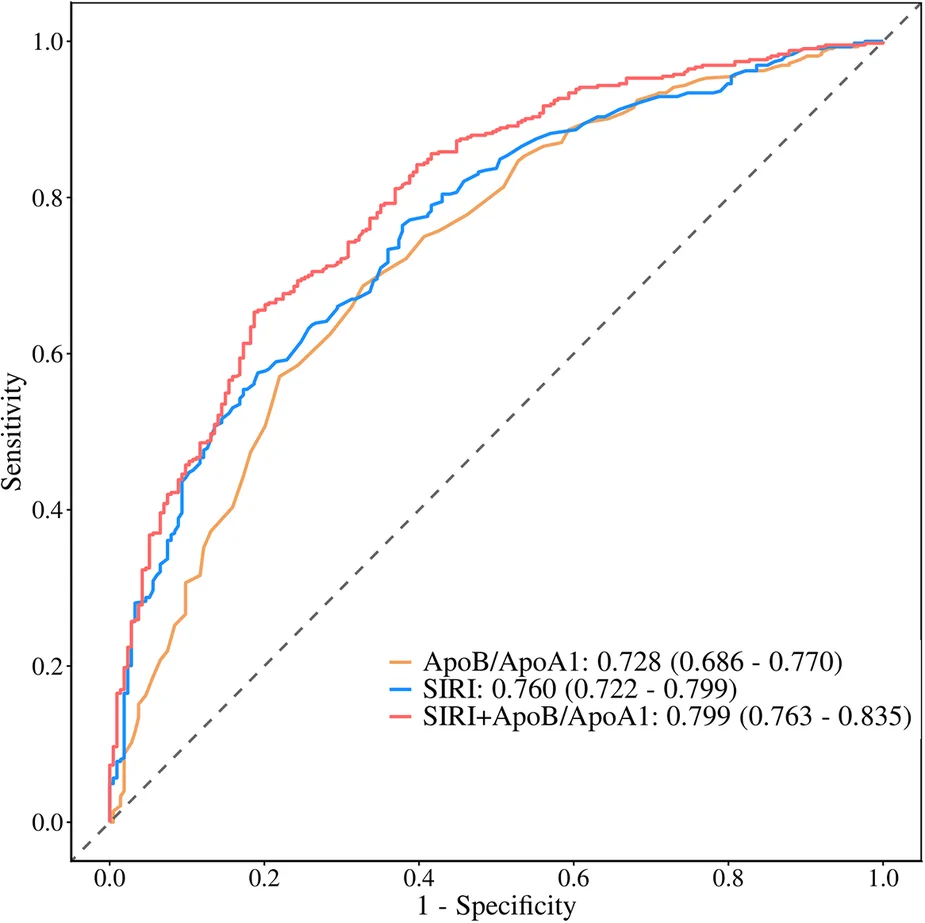
ROC Curves Evaluating the Presence of Atrial Fibrillation Based on SIRI, ApoB/ApoA1 (Separate and Combined Models).

### 3.5 Mediation analysis

A Bootstrap procedure of 5,000 samples was performed to generate confidence intervals, with covariate adjustment as in Model 3. The mediation analysis indicated that SIRI partly mediates the negative association between ApoB/ApoA1 and AF. Figure 3 depicts the indirect association through SIRI, which accounted for 28.1% of the total statistical effect. (indirect effect *β*Ind = −0.137, *P* < 0.001). The overall effect (*β*Total = −0.489) and the direct effect (*β*Dir = −0.352) of ApoB/ApoA1 on AF remained significantly negative.

### 3.6 ROC curve results

We performed a comparative evaluation of ApoB/ApoA1, SIRI, and their combination for classifying AF presence (Table 3). The ROC analysis (Figure 4) showed AUCs of 0.728 for ApoB/ApoA1, 0.760 for SIRI and 0.799 for the combined model. When contrasted with ApoB/ApoA1 alone, the ApoB/ApoA1 + SIRI combination achieved a significantly higher AUC (DeLong test, *P* < 0.001). Compared with SIRI alone, the integrated model also yielded a notably larger AUC (DeLong test, *P* = 0.002). The ApoB/ApoA1 + SIRI model exhibited the highest AUC, along with improved specificity, the lowest Brier score and the highest positive discriminative value, indicating optimal overall discriminative performance and calibration. This approach holds potential for exploratory clinical assessment and patient stratification, and may help reduce false-positive classifications in cross-sectional settings. However, these findings should not be overinterpreted as evidence of clinical diagnostic or screening utility; independent external validation and prospective confirmation are required before any routine clinical application can be considered.

**Table 3:** Discriminative capacity of SIRI, apoB/ApoA1 and their combination for AF presence.

Indicators	AUC	Sensitivity	Specificity	Youden	Cutoff	Accuracy	Brier score
ApoB/ApoA1	0.73	0.69	0.67	0.36	0.686	0.68	0.19
SIRI	0.76	0.76	0.62	0.38	0.577	0.72	0.18
ApoB/ApoA1 + SIRI	0.8	0.65	0.81	0.46	0.677	0.71	0.17

### 3.7 Subgroup analysis

To exclude the potential confounding effect of lipid-lowering therapy, we further performed a subgroup analysis stratified by medication use (Table 4). Subgroup analyses were conducted using the fully adjusted Model 3 framework. After dividing by the median, the link between higher SIRI and higher odds of AF was still seen in both people not taking lipid-lowering drugs (OR = 5.09, 95% CI: 3.02–8.58, *P* < 0.001) and those taking them (OR = 5.63, 95% CI: 1.22–25.91, *P* = 0.026). The effect did not differ between the two groups (*P* for interaction = 0.945). For ApoB/ApoA1, its inverse association with AF presence was strong in the non-drug group (OR = 0.17, 95% CI: 0.10–0.29, *P* < 0.001). In the lipid-lowering drug group, the protective trend remained but did not reach statistical significance (OR = 0.33, *P* = 0.104). However, the interaction was not significant (*P* for interaction = 0.370). Overall, these associations were not substantially modified by lipid-lowering drug use.

**Table 4:** Subgroup analysis of the associations of SIRI and ApoB/ApoA1 with AF presence, stratified by lipid-lowering drug use.

Indicator	*n* (%)	< Median (0.61)	> Median (0.61)	OR (95% CI)	*P*	*P* for interaction
ApoB/ApoA1						
All patients	638 (100.00)	248/300	176/338	0.19 (0.12 ∼ 0.31)	<0.001	—
LipidLoweringDrugUse	—	—	—	—	—	0.370
No	516 (80.88)	191/233	142/283	0.17 (0.10 ∼ 0.29)	<0.001	—
Yes	122 (19.12)	57/67	34/55	0.33 (0.09 ∼ 1.25)	0.104	—

	*n* (%)	< Median (0.99)	> Median (0.99)	OR (95% CI)	*P*	*P* for interaction
SIRI						
All patients	638 (100.00)	156/315	268/323	4.83 (3.01 ∼ 7.77)	<0.001	—
LipidLoweringDrugUse	—	—	—	—	—	0.945
No	516 (80.88)	124/261	209/255	5.09 (3.02 ∼ 8.58)	<0.001	—
Yes	122 (19.12)	32/54	59/68	5.63 (1.22 ∼ 25.91)	0.026	—

## 4 Discussion

To our knowledge, this study is the first clinical investigation to comprehensively describe the associations among ApoB/ApoA1, SIRI and AF prevalence. We found that SIRI, an inflammatory-immune index, was markedly higher in patients with AF. Further mediation analysis showed that SIRI statistically mediated the association between ApoB/ApoA1 and AF prevalence, suggesting that SIRI may act as a statistical intermediary in this link. These findings provide initial evidence that SIRI statistically correlates with AF presence. From a clinical perspective, these results suggest that inflammation associated with ApoB/ApoA1 may contribute to the pathophysiology of AF. Although abnormal lipid metabolism may be associated with AF, this link may be partially accounted for by inflammatory mechanisms.

The relationship between abnormal blood lipid levels and atrial fibrillation (AF) has long been controversial among researchers. While higher total cholesterol (TC) and low-density lipoprotein cholesterol (LDL-C) have been regarded as key AF risk factors (11, 12), some researchers have challenged this view, describing a cholesterol paradox (13, 14). In recent years, growing evidence indicated that lower LDL-C levels are associated with an increased AF risk (15, 16). Our research results are consistent with this newly emerging trend reported in existing literature. Specifically, we observed significantly lower concentrations of triglycerides (TG), total cholesterol (TC), low-density lipoprotein cholesterol (LDL-C), high-density lipoprotein cholesterol (HDL-C), apolipoprotein B (ApoB) and apolipoprotein A1 (ApoA1) in participants diagnosed with atrial fibrillation compared with non-AF patients.

The ApoB/ApoA1 ratio has substantial value in predicting CAD and metabolic syndrome. Its applicability was supported by numerous cardiovascular observational studies. Lu et al. identified this ratio as a key predictor of coronary disease in overweight and obese individuals (17). Higher ApoB/ApoA1 values have also been linked to subclinical atherosclerosis and unstable plaque burden (18). Bodde and colleagues reported an association between the ratio and the first STEMI event, although it did not predict subsequent MACE during follow-up (19). In adolescents with metabolic syndrome, Lee et al. found significant associations between the ratio and BMI, waist circumference, waist-to-hip ratio, and abdominal fat area, suggesting predictive value in that population (20). More recently, observational data from a Chinese cohort indicated that a lower ApoB/ApoA1 ratio was linked to AF in both men and women (5). ApoB is a major LDL component and a precursor of atherogenesis, reflecting the number of lipoprotein particles that can promote vascular disease (21). Several scholars have explored the relationship between ApoB and AF. Some studies report a negative association that is independent of gender and statin use, highlighting a complex interplay that warrants further study (22). Apart from lipid-related effects, ApoB has been shown to be a strong, independent predictor of inflammatory markers such as IL-6 and CRP in certain populations—e.g., postmenopausal overweight/obese women (23). Further work suggests that reduced ApoB might be linked to inflammation, whereas higher ApoB could represent a potential therapeutic target to mitigate obesity-associated inflammation (24). Taken together, these data raise the possibility that lower ApoB could initiate or sustain inflammatory pathways contributing to AF. ApoA1 plays a central role in HDL synthesis and participates in LCAT activation; HDL is regarded as protective against atherosclerosis and possesses anti-inflammatory, antioxidant and anti-thrombotic properties (25–28). Additionally, oxidation of tryptophan residues in ApoA1 may promote inflammatory responses (29–31). Evidence linking ApoA1 to AF remains limited, but a study in statin-naïve Chinese individuals reported that lower ApoA1 levels were significantly associated with AF in both sexes, supporting a link between ApoA1 status and AF risk (32). These studies verify that ApoA1 and ApoB are both strongly associated with inflammatory status. Consistent with their observations, our study demonstrated a concomitant reduction in ApoA1 and ApoB levels in patients with atrial fibrillation.

Inflammatory signals derived from peripheral blood cells significantly influence the development and persistence of AF, making CBC-derived markers important intervention targets. Various blood cell indicators including neutrophils, lymphocytes, platelets and monocytes are widely adopted to judge prognosis when dealing with inflammatory illnesses. Among all those biomarkers, the systemic inflammation response index, also known as SIRI, usually shows better predictive performance than many traditional indicators when judging disease development and clinical end events. Studies conducted across various patient groups have confirmed that the SIRI can stratify patients with AF into different risk categories and assist doctors to make personalized treatment plans. Previous research reports that SIRI serves as a convenient means to screen people with high AF risks at an early stage (33, 34). Several studies have reported that patients with post-operative atrial fibrillation (POAF) exhibit elevated SIRI values compared with control subjects (35, 36). A recent large-sample study including over 10,000 participants revealed that after adjustment for several peripheral blood inflammatory markers, there exists a clear positive correlation between SIRI levels and atrial fibrillation (37). Our results indicate that a lower ApoB/ApoA1 ratio corresponds to an elevated inflammatory state, which was in turn linked to the presence of AF. This association leads us to hypothesize that the concurrent reduction in both ApoB and ApoA1—and the consequent loss of their anti-inflammatory and antioxidant functions—may be involved in the pathophysiology of AF-related atrial remodeling. However, the precise mechanisms remain to be elucidated (38–40).

Our study suggests that including SIRI and ApoB/ApoA1 in atrial fibrillation exploratory clinical assessment models may be useful for identifying individuals with AF. The combined model incorporating ApoB/ApoA1 and SIRI demonstrated the strongest discriminative performance, achieving the highest area under the receiver operating characteristic curve (AUC), enhanced specificity and positive predictive value, and the smallest Brier score. These metrics show the combined model can tell AF patients apart more accurately and produce more reliable classification estimates. Besides, this model cuts down false positive readings without lowering discriminative accuracy, highlighting its potential utility for preliminary patient stratification in research and exploratory clinical evaluation. However, we emphasize that the present findings are based on a cross-sectional cohort; therefore, any claim regarding clinical utility for diagnosis, screening, or risk stratification requires independent external validation and prospective longitudinal studies before it can be considered for routine clinical implementation.

Our subgroup analyses show that for ApoB/ApoA1, the inverse association with AF was statistically significant in the non-drug group (OR = 0.17, 95% CI: 0.10–0.29, *P* < 0.001). In the drug group, the effect pointed in the same protective direction but did not reach significance (OR = 0.33, *P* = 0.104), likely due to the smaller number of patients (*n* = 122). The interaction between ApoB/ApoA1 and drug use was not significant (*P* = 0.370), still suggesting that the association is not substantially modified by lipid-lowering therapy.

Several additional limitations should be acknowledged. First, the retrospective design and the single-center, relatively small sample size limit the generalizability of our findings and our ability to control for all unknown confounding factors. Second, we only used baseline ApoB/ApoA1 and SIRI levels, without investigating their dynamic changes over time or analyzing the potential effects of clinical treatments. Due to the cross-sectional nature of the study, the temporal sequence cannot be ascertained. The possibility that AF itself induces the “lipid paradox,” leading to decreased apolipoprotein B and ApoA1 levels and consequently a lower ApoB/ApoA1 ratio and higher SIRI levels, cannot be excluded. Third, common inflammatory markers such as hs-CRP and IL-6 were not routinely measured, precluding a comparison between SIRI and these well-established classical inflammatory markers. Fourth, although all subjects underwent 24-hour Holter monitoring, brief AF episodes outside the monitoring window could still be missed; therefore, long-term cardiac monitoring studies are needed in the future to completely exclude missed AF. Finally, to clarify the causal sequence and support the core findings of this study, long-term prospective studies, or large-scale multi-center controlled trials incorporating repeated biomarker measurements and subgroup analyses stratified by AF subtypes, are still required.

## 5 Conclusion

Elevated SIRI and reduced ApoB/ApoA1 were significantly associated with higher odds of AF. The statistical mediation effect of SIRI suggests a potential interrelationship among these variables, although this should be interpreted as statistical interdependence rather than causality. The combined model outperformed all others in discriminative performance, offering a practical exploratory tool for patient stratification within a cross-sectional setting. However, independent external validation and prospective confirmation are mandatory, and its clinical diagnostic or screening utility remains to be established.

## Data Availability

The original contributions presented in the study are included in the article, further inquiries can be directed to the corresponding authors.
