# Correction: The inflammatory marker SIRI acts as a statistical mediator between ApoB/ApoA1 ratio and atrial fibrillation, and combined biomarkers show higher discriminative ability for the presence of atrial fibrillation than single indicators

**DOI:** 10.3389/fcvm.2026.1968329

**Published:** 2026-09-02

**Authors:** Chenyang Zheng, Meixue Jia, Bin Ning

**Affiliations:** Department of Cardiovascular Medicine, Anhui Medical University Affiliated Fuyang People’s Hospital, Fuyang People’s Hospital, Fuyang, Anhui, China

**Keywords:** ApoB/ApoA1, atrial fibrillation, inflammation, lipid metabolism, SIRI

The Data Availability statement was erroneously given as:

The datasets presented in this study can be found in online repositories. The names of the repository/repositories and accession number(s) can be found in the article/Supplementary Material.

The correct Data Availability statement is:

The original contributions presented in the study are included in the article, further inquiries can be directed to the corresponding authors.

The original version of this article has been updated.

